# Recent Theoretical and Experimental Progress in Circularly Polarized Luminescence of Small Organic Molecules

**DOI:** 10.3390/molecules23123376

**Published:** 2018-12-19

**Authors:** Naibo Chen, Bo Yan

**Affiliations:** 1 Department of Applied Physics, School of Science, Zhejiang University of Technology, Hangzhou 310023, China; chennb@zjut.edu.cn; 2 Collaborative Innovation Center for Information Technology in Biological and Medical Physics, Zhejiang University of Technology, Hangzhou 310023, China

**Keywords:** circularly polarized luminescence, small organic molecules, chirality

## Abstract

Small organic molecules (SOMs) with fascinating chiroptical properties have received much attention for their potential applications in photoelectric and biological devices. As an important research tool, circularly polarized luminescence (CPL) provides information about the chiral structures of these molecules in their excited state, and has been an active area of research. With the development of the commercially available CPL instrumentation, currently, more and more research groups have attempted to enhance the CPL parameters (i.e., quantum yield and dissymmetry factor) of the chiral SOMs from all aspects. This review summarizes the latest five years progresses in research on the experimental techniques and theoretical calculations of CPL emitted from SOMs, as well as forecasting its trend of development.

## 1. Introduction

Chirality is a property of asymmetry that has been widely studied in the areas of physics [1,2], chemistry [3,4] and biology [5,6]. Molecules having a non-superimposable mirror image are dissymmetric or chiral. They have the ability of interacting differently with left- and right-handed circularly polarized light, and can be utilized to study a wide variety of phenomena. Circularly polarized luminescence (CPL) is one of the chiroptical phenomena originating from chiral luminescence. Different from circular dichroism (CD) defined as the difference between the absorption of left- and right-handed circularly polarized light that provides the structural information about the electronic ground state, CPL gives the complementary information about the chiral characteristics of the excited state. Up to now, CPL has been used in many areas, such as three-dimension optical displays [7], optical information storage and processing systems [8], optical quantum information [9], molecular photoswitches [10], spintronics-based devices [11], biological probes and signatures [12], CPL lasers [13], enantioselective CPL sensors [14], promote asymmetric photochemistry [15], or light-emission systems for asymmetric photosynthesis [16].

Chiral molecules with efficient CPL are very useful for bio-sensing, bio-imaging and optoelectronic applications, such as probing biomacromolecular targeting events by reading the change of CPL signal, and devices for stereoscopic optical information processing, display and storage [17,18,19]. Before application, however, two key parameters have to be simultaneously optimized. One is the quantum yield (ϕ_F_) defined as the ratio of the number of photons emitted to the number of photons absorbed. The possible value is between 0 and 100%. Another is the dissymmetry factor (g_lum_) defined by the equation of g_lum_ = 2ΔI (λ)/I (λ) [20]. Here, ΔI (λ) and I (λ), both being a function of wavelength, represent the emission circular intensity differential and the total intensity of the left- and right-handed circularly polarized components, respectively. Therefore, the possible g_lum_ values are within the range of −2 (completely right emission) to +2 (completely left emission), while 0 corresponds to an unpolarized emission. Moreover, g_lum_ also can be defined theoretically as 4|*m*|·|*μ*|·cos*θ*/(|*m*|^2^ + |*μ*|^2^) [21], where *m* and *μ* are the magnetic and electric transition dipole moments, respectively, and *θ* is the angle between *m* and *μ*. The large |g_lum_| values are only expected for *m*-allowed and *μ*-forbidden transitions. Nowadays, the values of |g_lum_| achieved from chiral lanthanide complexes were much larger because of their *f*-*f* Laporte forbidden transitions [22], and the largest value had been to 1.38 [23]. Due to the involved metal-centered electronic transitions, however, the ϕ_F_ values of these complexes are usually small, hindering their CPL applications. Therefore, more efforts have been devoted to chiral organic molecules for their high emission quantum yields, easy processing, tunable emission wavelengths, easy structure modification, and potential applications in new generation display materials [24].

Small organic molecules (SOMs) exhibit smaller densities, lighter weights and excellent organic-solvent solubility. With the absence of transition metals, CPL spectroscopy based on SOMs has attracted considerable attention during the last several decades. For example, CPL emitters based on SOMs (ϕ_F_ = 74%, |g_lum_| = 1.3 × 10^−3^) have been used to fabricate circularly polarized organic light emitting diodes (CP-OLED) exhibiting external quantum efficiency of 9.1% in 2016 [25]. Unfortunately, SOMs always exhibit much smaller levels of CPL (|g_lum_| ≈ 10^−5^–10^−3^) [26], because the molecular sizes are much smaller than the helical pitch of circularly polarized light, and the *μ*-allowed transitions induce the much larger value of I (λ) than that for lanthanide complexes. Only a small number of SOMs can display relatively high performance both in terms of ϕ_F_ and |g_lum_|. Obviously, new structural designs for chiral SOMs, combining together CPL activity and emission, are needed in order to develop usable, smarter, and better CPL characteristics. In this review, we enumerate the theoretical and experimental progress in CPL of chiral SOMs in the latest five years. We also try to analyze the existing situation and prospect the future research direction to help researchers design and apply SOMs to fabricate CPL active materials or devices.

## 2. Progress on Theoretical Calculations

In order to have more and more SOMs suitable for practical applications, the design of chiral systems endowed with a high |g_lum_| is desirable. To accomplish this task, quantum mechanical calculations can guide the rational design of efficient CPL by providing insight in the chirality of the excited state, although such theoretical results still scarce in the literatures. Pritchard and Autschbach [27] firstly used density functional theory (DFT) to compute Franck-Condon vibrationally resolved absorption, emission, and CPL bands corresponding to the lowest-energy n→π∗ transition of the small chiral ketones, d-camphorquinone, (*S,S*)-*trans*-β-hydrindanone, and (1*R*,5*S*)-*cis*-β-hydrindanone, for comparison with well-resolved experimental spectra. Pecul and Ruud [28] also carried out a series of DFT computations of CPL spectra of organic ketones. Moreover, without taking the vibronic contributions into account, CPL spectra can be calculated through the following equations [27,29] in which dipole (*D*_0*m*_) and rotational strengths (*R*_0*m*_) are evaluated in the excited state geometry optimized by time-dependent density functional theory (TD-DFT) calculations [30]:

(1) $$I\;=\;\frac{4E^{3}\rho \left(E\right)}{3\cdot \hbar^{4}\cdot c^{3}}D_{0m},$$

(2) $$\Delta I\;=\;\frac{\;16E^{3}\rho \left(E\right)}{3\cdot \hbar^{4}\cdot c^{3}}R_{0m},$$

where *ħ* is the reduced Planck′s constant, *c* is the speed of light, and *ρ(E)* is a Gaussian band shape in terms of energy. The above equations can give CPL spectrum for each emitting molecule, but such theoretical spectra are not easy to be used to compare with experimental spectra. The reasons include that it is difficult to know how many molecules are involved in the emitting state, and the collected radiation depends on instrumental characteristics. From the above equations, the dissymmetry factor can be calculated as [31]:

(3) $$g_{\mathrm{lum}}\;=\;\frac{4R_{0m}}{D_{0m}}$$

In 2014, Abbate et al. [32] prepared four different hexahelicenes, namely 5-azahexahelicene, hexahelicene, 2-methylhexahelicene, and 2-bromohexahelicene. Each type of experimental CPL spectrum was compared with the corresponding theoretical spectrum. In order to calculate and interpret CPL spectra, molecular structures were optimized in their first excited states at CAMB3LYP/TZVP level and transition energies, dipole and rotational strengths were calculated by TD-DFT. Calculations showed a good correspondence with experimental data, although all of them had weak CPL spectra. According to the results, CPL spectra were significantly different in the four cases. 5-aza-hexahelicene exhibited the largest CPL, and in the other cases, CPL was smaller.

In 2015, Crawford et al. [33] carried out the first equation-of-motion coupled cluster singles and doubles (EOM-CCSD) calculations of CPL rotatory strengths for comparison to the results from TD-DFT (B3LYP and CAM-B3LYP) using a series of eight chiral ketones as a test set. For most of the cases, EOM-CCSD and CAM-B3LYP exhibited relatively good agreement. They also compared theoretical and experimental CPL spectral data for two β,γ-enones, (1*R*)-7-methylenebicyclo[2.2.1] heptan-2-one and (1*S*)-2-methylene bicyclo[2.2.1]heptan-7-one, which exhibited two conformers on the first excited-state potential energy surface. EOM-CCSD and CAM-B3LYP provided closer agreement with experiment for both dipole and rotatory absorption strengths, while B3LYP yielded better agreement for the corresponding dissymmetry factor due to cancellation of errors.

In 2016, Villani et al. [34] studied two thia-heterohelicenes (a hetero [4]-helicene and a hetero [6]-helicene) of different length by CPL. In order to predict CPL spectra to determine the origin of the observed bands, molecular structures were optimized in their first excited states by TD-DFT calculations at the CAM-B3LYP/TZVP level and transition energies, dipole strengths and rotational strengths were calculated using TD-DFT. The calculated |g_lum_| for [6]-helicene was about 1 × 10^−2^, agreed well with the experimental one, but for [4]-helicene, the |g_lum_| value (6 × 10^−2^) was much larger than the experimental value since the enantiomeric excess was not under full control during the CPL measurement, where degradation and partial racemization occurred.

Boron dipyrrin derivatives (BODIPY, 4,4-difluoro-4-bora-3a,4a-diaza-s-indacene) are famous fluorophores with high absorption coefficients and high quantum yields. In 2016, Di Bari et al. [35] studied the circularly polarized emission properties of two quasi-isomeric BODIPY “DYEmers” differing in the position of the aryl-aryl junction. The calculation procedure was based on the optimization of the molecule in its first singlet excited state geometry, and on the evaluation of the excited states obtained thereof. The calculation for compound 1 was run with TD-DFT at the M06-2X/def2-TZVP level in vacuo, and the predicted sign of the CPL band was in agreement with the experimental one. The computed value for |g_lum_| was very close to the experimental value (5.6 × 10^−^^3^ vs. 3.8 × 10^−^^3^). For compound 2, the CPL spectrum was very weak (4.0 × 10^−^^4^). The wrong sign for the CPL band was occurred using TD-DFT, but SCS-CC2 or a DFT functional with full exact exchange provided the correct sign.

In 2016, Santoro et al. [36] reported their calculations of CPL for the lowest excited state of hexahelicene, 2-methylhexahelicene, 2-bromohexahelicene, and 5-azahexahelicene. All the CPL spectra were computed by applying TD-DFT combined with Adiabatic Hessian and Vertical Hessian models. Both Duschinsky and Herzberg-Teller effects were considered. 5-azahexahelicene exhibited the simplest CPL spectrum with a clear positive sign. Computed spectra for 5-azahexahelicene were similar with any of the three adopted models. The experimental CPL spectrum of 2-bromo-hexahelicene was dominated by noise because of the very low emissive intensity of this molecule. But the experimental spectrum suggested a negative signal, correctly predicted by the computations. However, for hexahelicene and 2-methylhexahelicene, the computed peaks appeared less resolved than the experimental ones.

In 2018, Mori et al. [37] aligned two hexahelicenes in various orientations and examined by theoretical calculations to predict the best chiroptical performance for X-shaped and S-shaped double hexahelicenes. The TD-DFT calculations were performed at the M06-2X/def2-TZVP level. Excited-state structures were optimized by the time-dependent, second-order approximate coupled-cluster singles and doubles model at the RI-CC2/def2-TZVPP level. The theoretical results showed the sign and relative intensity of CPL among the single and double helicenes. X-shaped and S-shaped double hexahelicenes could produce more than twice intensified CPL. This conclusion was proven by the experimental results. Combined with the theoretical and experimental results, it was convenient to find how the molecular symmetry and the alignment of chiral elements determine the CPL responses by manipulating electric and magnetic transition dipole moments of the molecule.

The current studies indicate that the theoretical calculation can be used not only to explain the CPL experimental phenomena, but also to provide a reliable guidance for designing novel advanced SOMs with good CPL responses.

## 3. Progress on Experimental Researches

### 3.1. Improvement on the CPL Measurement Instrument

CPL spectroscopy is used for characterizing chiral emissive chromophores and is an offset of a branch of spectroscopy known as CD spectroscopy measuring the chirality of the absorption spectrum. CPL of SOMs has been observed for electric dipole allowed π→π* transitions either in molecules with intrinsically chiral fluorophores (such as helicenes and helicene-like molecules), in chirally perturbed chromophores (such as monomeric BODIPYs), or in exciton-coupled systems (such as functionalized binaphthyls or BODIPYs dimers). However, unlike CD spectroscopy for which commercial instruments have been available for more than 50 years, the measurement of CPL has mainly performed with homebuilt apparatus by a limited number of research groups [31,38,39,40,41]. Nowadays, CPL spectra are usually performed on a commercial instrument, such as JASCO CPL spectrofluoropolarimeter, OLIS DSM, at the room temperature on the basis of the Stokes-Mueller matrix approach. Thus, in the last five years, the CPL research experienced a rapid progress particularly in the study focused on SOMs, due to the much easier access to CPL instrument.

Taking the JASCO CPL 200 spectrometer as an example, the basic composition and principle of the modern chiroptical spectrophotometer [42] can be introduced simply as follows. Without using a laser, a white light from the Xe lamp passes through the flat-field grating monochromator (VF-P0240, Shinkukogaku Co., Ltd., Tokyo, Japan), and is converted into monochromatic light. Then it is collected by the focusing lens, and strikes a right-angle prism (RPSQ-25-10H, SIGMAKOKI Co., Ltd., Tokyo, Japan), deviating the beam normal to the incident face by 90°. After traversing the depolarizer (PDH15, Bernhard Halle Nachfl. GmbH, Berlin, Germany), the light is converted into unpolarized radiation and collected by the following condenser lens to strike the sample. The emitted light from the sample is collected by the collimator lens (SLSQ-25B-120P, SIGMAKOKI Co., Ltd.), and then falls on the photo-elastic modulator (PEM) driven by a piezoelectric transducer to oscillate at 50 kHz. With respect to the horizontal plane, the PEM is fixed at 0° to give the relative phase to the orthogonal component of the transmitted light, while the following analyzer is angled in the optical axis at 45°. Emerging from the analyzer, the light successively passes through the secondary right-angle prism and collimator lens. After impinging on the double-prism monochromator, the light is monochromatic, and is collected by a collimating lens. Then the light strikes a photomultiplier (PM). The output from PM is a DC photocurrent superimposed by modulated AC components. This signal is converted into a voltage by a transimpedance preamplifier. The signal processing is set up to record the ratio of the AC to the DC signals, and are transmitted to a PC.

It can be seen that the optical configuration of the commercial instrument is similar to those of homemade ones except for the fluorescence monochromator being set parallel to the azimuth of the excitation light. This instrument design [42] opens a new field of solid-state CPL measurements applicable to samples in liquid phases, mesophases, and condensed phases because the samples can be placed on a horizontal plane. Additionally, two prism monochromators are used to measure sharp CPL peaks at a high resolution, especially for the visible and the ultraviolet region. Both the emission and excitation monochromators are equipped with continuously variable slit drives, which allow for an appropriate wavelength and band width selection. Moreover, this instrument includes a pulse motor-driven sample rotation holder and a 100 kHz lock-in amplifier to achieve the linearly polarized luminescence measurement to obtain the true CPL signal. With the holder, the maximum possible volume of sample can be excited without excitation light reflecting or refracting off the edges of the sample-air interface, and the maximum possible amount of light can be collected by the collimator lens to enhance the sensitivity. For the light intensity sharply decreases below 250 nm, however, the CPL spectrophotometer still has a limited spectral region of 250–800 nm. Therefore, in future there will be a necessity of a specific improvement that will enable measurements down to 190 nm with high sensitivity.

### 3.2. Improvement on the CPL Experimental Results

Molecules with CPL properties can be classified into four types [43]: central chiral molecules, axial chiral molecules, planar chiral molecules, and helical chiral molecules. Generally, central chiral molecules have one or more carbon atoms with four different groups attached. Each such tetrahedral carbon or other chiral center can be characterized by a letter *R* or *S*. Axial chirality arises when four groups sit in a non-planar arrangement, and can be classified as P (or *R*) and M (or *S*). Planar chirality can be regarded as a special case of chirality for two dimensions. It results from the arrangement of out-of-plane groups with respect to a chiral plane, and can be assigned as *R* or *S*. For helical chirality, a helix, propeller or screw can be twisted left (M) or right (P) around its axis.

#### 3.2.1. Central Chirality

Few molecules have intrinsically central chiral fluorophores. Therefore, embedding organic chromophores in a chiral matrix is one of the important strategies explored recently to increase the circular polarization. As shown in Table 1, naphthyl derivatives (**C1** and **C2**) possessing four 1- or 2-naphthyl groups introduced to the same chiral scaffold derived from tartaric acid were designed by Imai et al. [44]. Although both of them had the |g_lum_| value being in the range of 10^−3^, the trivial positional difference in the naphthyl substituent led to the sign inversion in CPL spectra, providing a simple method for switching the CPL signs just by introducing regioisomeric fluorophores. It suggested the organic CPL materials could be designed more freely, and their signs could be manipulated easily. A similar strategy was employed by Cheng et al. [45] to design compound (**C3**) using four chiral 1,2-diaminocyclohexane-based molecule incorporating 1,8-naphthalimide fluorophores with the high |g_lum_| value of 1.4 × 10^−2^. However, the reversed CPL signals only could be observed in the aggregated state.

Chiral distortion or modification incorporated into achiral dyes (BODIPY or pyrene) is another strategy to design molecules with central chirality. In the visible and near-infrared light region, such highly fluorescent dyes usually have the strong absorption and high quantum yield. By linking C_2_-symmetric chiral binaphthyl or 1,2-diamino-cyclohexane derivatives to them, molecules with central chirality can emit CPL efficiently. Therefore, a novel chiral BODIPY (**C4**) with twisted skeleton was synthesized by Nabeshima et al. [46] through oxidation of cyclic biphenyl units at the β positions. Using this method, the biphenyl carbons linked by cyclization becomes the asymmetric centers of sp^3^. Interestingly, the elongated π-conjugation and the twisted structure make it easy to exhibit a large fluorescence ϕ_F_ but a low |g_lum_| in the red region. Moreover, due to the emission band of pyrene-containing fluorophores is switchable between monomer and excimer emission, Ito, Imai and Asami et al. [47] synthesized the chiral pyrene-based *N*,*N*′-dipyrenyldiamine (**C5**), 4,6-*bis*(1-(pyren-1-ylamino)propyl)dibenzo-[b,d]furan to exhibit an unprecedented CPL switching behavior. According to the measurement results, dilute toluene solutions exhibited maximum CPL intensities in the monomer emission region, while saturated solutions exhibited a sign-inverted strong CPL in the excimer region. This phenomenon should be attributed to the emission of a strong CPL from a minor pyrene excimer with a rigid chiral environment.

It is obvious that molecules with central chirality are applicable to the future development of CPL-switchable luminophores used in security technologies and sensing devices. Compared to the chiral perturbation by embedding organic chromophores in a chiral matrix, linking chiral moieties to BODIPY or pyrene organic dyes usually resulted in larger ϕ_F_ values but lower g_lum_ values.

#### 3.2.2. Axial Chirality

Axial chirality refers to stereoisomerism resulting from the non-planar arrangement of two or four groups in pairs about a chiral axis. Among axially chiral compounds, biaryls, especially binaphthyl compounds, are one of the most important and useful compounds for monitoring steric effects and noncovalent interactions.

For axially chiral molecules, as shown in Table 2, most of the current researches focused on the influence of solvent on the CPL performance and carried out by the group of Imai. In the same solution of CHCl_3_ (1 × 10^−4^ M), 1,1′-binaphthalen-2,2′-diol (BINOL) (**A1**), its corresponding derivatives (**A2**) based on pyrene, and binaphthyl organic fluorophores (**A3**) had similar CPL performance [48,49]. Among them, **A2** corresponding menthylcarbonate had the largest ϕ_F_ (80.0%) and the highest|g_lum_| (1.2 × 10^−3^). According to the phenomena occurred in **A3**, the introduction of an alkenyl group as a π-conjugated substituent onto the binaphthyl backbone resulted in the blue-shifted CPL wavelength without inverting the CPL sign. It was different from the CPL sign inversion induced by the introduction of aromatic substituents onto the binaphthyl, and provided a new method for the synthesis of novel chiroptical luminophore systems. To further verify the effect of different organic solutions on the CPL sign, two open- and closed-type binaphthyl compounds (**A4** and **A5**) were studied in two solvents (CHCl_3_ solution and DMF solution) with the same concentration [50]. The values of |g_lum_| were close to those of **A1**–**A3**. Due to the Ar′-C-C-Ar″ dihedral angle between the phenanthrene rings and binaphthyl, the CPL sign of **A5a** with phenanthrene as the luminophore was inverted in DMF. But this inversion phenomenon didn’t emerge in **A4a**. It demonstrated that choosing proper substituents on the binaphthyl rings as luminophores was an efficient method to control the CPL sign in binaphthyl derivatives. Moreover, binaphthyl derivatives, π-conjugated 2,2′-diphenyl-4-biphenanthrol (VAPOL) (**A6**) [51] exhibited an efficient CPL properties (1.3 × 10^−3^) in CHCl_3_ solutions. Its ϕ_F_ decreased when the concentration increased, with values of 20% (1.0 × 10^−5^ M) > 16% (1.0 × 10^−4^ M) > 13% (1.0 × 10^−3^ M), and the emission wavelength value was greatly red-shifted as the concentration increased, with values of 376 nm (1.0 × 10^−5^ M) < 378 nm (1.0 × 10^−4^ M) < 391 nm (1.0 × 10^−3^ M). Different to 3,3′-diphenyl-2,2′-*bi*-1-naphthol (VANOL) exhibiting no CPL or PL, the ϕ_F_ values of optically active aryl fluorophores (**A7**) (VANOL hydrogen phosphate) and (**A8**) (VAPOL hydrogen phosphate) in CHCl_3_ solution were higher, and the CPL signs in solution state and in solid state were successfully controlled by modifying the internal axial chirality and the axial bonding position of the biaryl units [52]. Excitedly, in 2018, two new inherently chiral oligothiophenes (**A9** and **A10**) synthesized by Longhi et al. [53] based on the atropisomeric 3,3′-bithianaphtene scaffold showed the remarkable CPL characteristics, and the highest |g_lum_| values reached ~10^−2^ in the CHCl_3_ (1.0 × 10^−4^ M) solution.

Chiral materials can be studied in different solvents, and chiral behaviors in one or two media (organic solvents or solid state) have been reported. In highly polar media, especially in water, their chiroptical behaviors suffer from severe decay, hindering their practical applications. To further study the influence of various solvent states, signals of 2,2′-*bis*(diphenylphosphino)-1,1′-binaphthalene (BINAPO) (**A11**) [54], open-style 3,3′-*bis*(triphenylsilyl)-1,1′-*bi*-2-naphthol (**A12**) [55] and closed-style 3,3′-*bis*(triphenylsilyl)-1,1′-binaphthyl-2,2′-diyl hydrogenphosphate (**A13**) [55] were detected in the CHCl_3_ solution, PMMA-film-dispersed, and KBr-dispersed states, respectively. The ϕ_F_ values of **A11** were larger in both of KBr and PMMA (7%), while the |g_lum_| value was higher in CHCl_3_ (1.2 × 10^−3^). Both of the CPL wavelengths of **A12** and **A13** were red-shifted by introducing bulky C_3_-symmetrical triphenylsilyl groups into the binaphthyl unit. The CPL signals of them were opposite but were relatively larger in three solvents. This phenomenon of the sign inversion was attributed to the rotation of the C_2_-axis by the bulky groups. Similarly, the CPL sign of a binaphthylacetic acid organic luminophore (**A14**) [56] was successfully controlled by changing the solvent (CHCl_3_, CH_3_CN, DMF, MeOH, and PMMA film). The fluorescent modes and signs of CPL of binaphthyl-pyrene organic fluorophore (**A15**) [57] were successfully controlled by changing from a fluidic CHCl_3_ solution to a glassy poly(methyl methacrylate) solid PMMA film. It exhibited the negative CPL sign in the CHCl_3_ solution, and the positive CPL sign in the PMMA film. This finding may offer the choice of fluidic solution and glassy solid to control the CPL characteristics of multiple fluorophore molecules in addition to chiral pyrene-based fluorophores. In 2018, using a Newman-Kwart rearrangement reaction, the *O*,*O*-*bis*(*N*,*N*-dimethylthiocarbamate) (CPL-silent) was converted to the *S*,*S*-*bis*(*N*,*N*-dimethylthiocarbamate) (CPL-active, **A16**) at the peripheral positions of chiral 1,1′-binahphthalene-2,2′-diyl to design a turn-on type CPL-functioning simple molecular emitter [58]. **A16** exhibited clear near-mirror-image CPL signals in CHCl_3_ with the |g_lum_| value about 1.2 × 10^−3^. However, due to the unresolved interactions between the polymers and **A16**, CPL signals failed to occur in PMMA and ARTON films.

Compared to those organic solvents, emitting CPL efficiently in water was rarely reported. Therefore, two different types of amphipathic binaphthyl fluorophores were studied, the open-style **A17** [59] and the closed-style **A18** [59]. The dihedral angle of the binaphthyl unit could be used to control the CPL sign in water, although the values of |g_lum_| were low. They proved that the signs and wavelengths of CPL signals were significantly affected by the rotation tenability of the internal-axis binaphthyl fluorophore, even in water. In addition, a unique two-tandem fluorophoric molecular system (**A19**) [60] exhibited a CPL signal at ~410 nm with |g_lum_| of 5.0 × 10^−4^ and ϕ_F_ of 3.0% by chirality transfer from binaphthyl to two terthiophene subunits in the solid state. It seems that chirality transfer from chiral binaphthyl to achiral fluorophores is a new idea for the design of solid-state CPL materials.

Similar to the central chiral molecules, embedding organic chromophores in highly fluorescent (achiral) dyes is another important strategy to design efficient CPL emitters. The first example of a new structural design was O-BODIPY (**A20**) [61], achieving CPL from an inherently achiral organic chromophore. de la Moya’s group [62] also reported a new structural design (**A21**), in which the luminescent BODIPY chromophore (1,3,5,7,8-pentamethyl-2,6-diethyl-F-BODIPY) was orthogonally attached to an axially chiral 1,1′-binaphthyl unit (1,1′-*bi*(2-naphtol) (BINOL)). Although the measured |g_lum_| in solution was only 7.8 × 10^−4^, the quantum yield (48.0%) was large and the architecture was simple. Pescitelli et al. [35] measured CPL spectra of the enantiomeric pairs of two quasi-isomeric BODIPY DYEmers (**A22** and **A23**) endowed with axial chirality and dominated by the exciton coupling between the main π–π* transitions (550–560 nm) of the two BODIPY rings. Nakano′s group [63] synthesized chiral spiro polycyclic aromatic compounds with thiophene and/or thiophene *S*,*S*-dioxide. The synthesized chiral spiro compound (**A24**) exhibited CPL with a |g_lum_| value < 3.0 × 10^−3^. The work was the first example of CPL property of spiropolycyclic aromatic compounds with a spiro carbon atom. Ema et al. [64] synthesized a series of oligonaphthodioxepins (**A25–A30**), revealing a helically arranged octamer, **A27**, which showed intense CPL both in solution and in solid state. The fluorescence quantum yields in solution and in solid state were 90.0% and 22.0%, respectively, and the |g_lum_| values in solution and in solid state were 2.2 × 10^−3^ and 7.0 × 10^−3^, respectively. This is one of the highest solid-state CPL |g_lum_| values reported. The increased ϕ_F_ and |g_lum_| values were due to the rigidity, as well as to the fact that **A27** was a non-planar molecule. Moreover, **A27** was highly stable both chemically and stereochemically. In 2018, Imai et al. [65] synthesized three chiral rotatable oligonaphthyl fluorophors (**A31**–**A33**) through π-conjugation extension. With the number of naphthyl units increased from two to four, all the wavelengths of CPL signs exhibited in CHCl_3_ solution and PMMA were red-shifted, and all the quantum yields were increased. The values of ϕ_F_ in solution were higher than those in solid film state. For the value of |g_lum_|, it decreased with increasing the number of naphthyl units, but the effect of solvent was small.

It is obvious that axially chiral molecules usually result in lower |g_lum_| values, except for **A9** and **A10**. The fluorescence quantum yields of these compounds are varied in different solvents. Except naphthodioxepin oligomers, the maximum quantum yields were obtained from BINOL derivatives **A2** (ϕ_F_ = 80.0%) in CHCl_3_ and most of the |g_lum_| values were still in the region of 10^−3^–10^−4^. It may be due to the reason that the oscillation of axial chirality reduces the rigidity of chiral molecules in the excited state and influence the intensity of CPL. Therefore, designing a relatively rigid chiral skeleton is important to increase the |g_lum_| value in the future.

#### 3.2.3. Planar Chirality

The small molecules with planar chirality have found wide applications in asymmetric catalysis, chiral discrimination, and molecular devices, duo to their strong steric hindrance, tunable conformation, and excellent molecular recognition ability. [2.2]Paracyclophane, formed by stacking two adjacent benzene rings, is a unique aromatic compound. One interesting structural characteristics of the [2.2]paracyclophane skeleton is planar chirality produced by the proximal immobilization of benzene rings. Up to date, most of the researches focused on [2.2]paracyclophane.

As shown in Table 3, a propeller shaped macrocyclic compound (**P1**) was synthesized by Morisaki et al. [66]. Starting from 4,7,12,15-tetrabromo[2.2]paracyclophane, the macrocycle obtained through coupling reactions by a diastereomer method exhibited a large ϕ_F_ of 45.0% and a high |g_lum_| of 1.1 × 10^−2^. This research group also investigated the chiroptical properties of planar chiral [2.2]paracyclophane-based through-space conjugated oligomers. All the optically active compounds (**P2**–**P5**) [67] exhibited relatively large ϕ_F_ values and high |g_lum_| values. Moreover, based on 4,7,12,15-tetrabromo[2.2]paracyclophane, they designed and synthesized optically active X-shaped π-conjugated dimers (**P6–P8**) [68], arising from two stacked p-phenylene-ethynylenes functionalized by benzene, naphthalene, and anthracene. Among these compounds, the naphthyl-containing dimer **P7** achieved the highest |g_lum_| of 1.7 × 10^−3^, and the largest ϕ_F_ of 78.0%. Then they reported optically active Frechet-type dendrimers (**P69**) [69] with a relatively high |g_lum_| of 1.5 × 10^−3^. Containing an X-shaped conjugated core with the planar chiral [2.2]paracyclophane moiety, this compound had two p-phenylene-ethynylenes stacked at the central phenylene units. In 2018, they used chemoselective Sonogashira-Hagihara coupling to achieve another optically active X-shaped compound (**P10**) [70], composed of two different p-electron systems stacked at central aromatic rings. This compound also exhibited a high |g_lum_| of 1.7 × 10^−3^, and a value of ϕ_F_ larger than 70.0%. Interestingly, they synthesized cyclic compounds with extended π-conjugated systems still based on planar chiral [2.2]paracyclophane [71]. The |g_lum_| values of 3Ph (**P11**), 5Ph (**P12**), and 7Ph (**P13**) were 1.4 × 10^−3^, 1.2 × 10^−3^, and 1.0 × 10^−3^, respectively. And the |g_lum_| values of 3PhC (**P14**), 5PhC (**P15**), and 7PhC (**P16**) were 1.3 × 10^−2^, 1.0 × 10^−2^, and 0.75 × 10^−2^, respectively. Obviously, the values of |g_lum_| for **P14**–**P16** were higher than those for **P11**–**P13**, but the values of ϕ_F_ for the former were smaller than those for the latter. Moreover, using left- and right-handed double helical structures, a new planar chiral *bis*-(para)-pseudo-*ortho*-type 4,7,12,15-tetrasubstituted [2.2]paracyclophane (**P17**) [72] was synthesized by them. This compound had a large emission (ϕ_F_ = 62.0%) and an excellent chiroptical properties (|g_lum_| = 1.6 × 10^−3^). DPh1 (**P18**) and DPh2 (**P19**) synthesized by them [73] were optically active phenylethene. Due to the planar chiral, rigid, and stable 4,7,12,15-tetrabromo[2.2]paracyclophane scaffold, **P18** exhibited excellent CPL profiles in the diluted solution, while **P19** had good CPL profiles in the aggregation state. At the same time, they synthesized enantiopure phenylene-ethynylene dimers with pyridine groups [74]. In the (Sp)-N-Ph (**P20**) system, a strong negative signal at around 420 nm (|g_lum_| = 1.2 × 10^−3^) was derived from the same structure with (Sp)-N-H (**P21**). But a weak positive signal at around 530 nm (|g_lum_| = 2.5 × 10^−4^) might be derived from the π-π interaction of phenylene-ethynylene moieties. According to the above results achieved only by using carbon and hydrogen atoms, the researchers believed that the [2.2]paracyclophane unit would be a general component for the construction of advanced optical materials with multiple functions.

Yamanoi et al. [75] developed an easily accessible synthetic route to provide disilane-bridged cyclophanes via Pd-catalyzed double arylation. Some of the compounds displayed the inversion at room temperature, but this was controlled by altering the phase (solution vs solid), the bulkiness of substituent, and the inclusion into a host molecule. The CPL properties of optically active molecule (**P22**) were comparable to those of reported low-molecular-weight organic molecules. These findings of multifunctional tetrasila[2.2]cyclophanes provided a new molecular design strategy for functional organosilanes, donor-acceptor systems, and planar chiral systems. Isobe et al. [76] reported the chirality of nanoscale cylinders (“cylinder chirality”) resulted in chirality of larger dimensions in the form of a double-helix assembly (**P23** and **P24**). Cylinder chirality in solution gave rise to a large dissymmetry factor (10^−2^) of metal-free entities in circular polarized luminescence. This study unequivocally demonstrates that rigid nanoscale cylinders, including SWNT congeners, are promising CPL emitters that can fulfill such apparently paradoxical requirements.

Compared with the axially chiral molecules, planar chiral molecules usually exhibit much larger ϕ_F_ and higher |g_lum_| values, and their CPL wavelengths are in the range of 400–500 nm. The maximum quantum yield was obtained from **P13** (ϕ_F_ = 88.0%) and the maximum dissymmetry factor was obtained from **P23** (|g_lum_| = 1.5 × 10^−2^). Maybe, planar chiral molecules are the representatives of SOMs with good CPL performance.

#### 3.2.4. Helical Chirality

The most important examples of helical-shaped small molecules are helicenes and their derivatives. Helicene is a compound comprised of planar aromatic molecules such as benzene rings. When many benzene rings bond together, a 3D screw-shaped helical structure is formed to avoid clashing of the terminal rings known as steric hindrance. Ever since Newman and co-workers successfully synthesized optically active hexahelicene in 1956, helicenes have fascinated chemists, and thus many synthetic strategies and methods have been devised for the preparation of helicenes in appreciable amounts. Enantiomeric helicenes also exerted large CPL dissymmetry owing to the strong helical distortion of π-conjugation systems. The screw-shape of helicene framework is the main reason of its chirality although helicenes do not have any asymmetric carbon centers. Chiral helicene has been proved a valuable structural design for the development of CPL of SOMs.

With respect to their structure, helicenes can be divided into three main categories. Carbohelicenes consist solely of *ortho*-fused benzene rings. Heterohelicenes have one or more heteroatoms incorporated in their structure. Finally, the helicene-like compounds are not fully aromatic compounds but possess the helical twisted shape. Geometrical properties define whether a molecule is a helicene or not, and also determine which class a helicene belongs to: carbohelicene, heterohelicene or helicenoid(helicene-like) structure.

Carbohelicenes are generally incorporated a helical, distorted, conjugated polyaromatic ortho-fused benzenoid rings system, which is a fundamental molecular characteristics of this class of compounds. They are named as [*n*]helicenes, where *n* represents the number of rings forming a helix in the ortho-fused fashion. The structures **H1**–**H6** in Table 4 are carbohelicenes, respectively. Sakai et al. [77] designed and synthesized a 1,2-dialkylquinoxaline-fused [7]carbohelicene (**H1**) by asymmetrically introducing two alkyl chains onto the quinoxaline unit. The spectra of **Hl** were significantly red-shifted compared to those of [7]carbohelicene. The values of ϕ_F_ and |g_lum_| were 25.0% and 4.0 × 10^−3^, respectively. Also, they designed and synthesized maleimide-substituted [5]carbohelicene (**H2**) and methoxy-substituted HeliIm (**H3**) by electron-withdrawing maleimide and electron-donating methoxy [78]. The ϕ_F_ value of **H2**, 37.0%, was larger than that of **H3**, 22.0%, but the two examples had the similar |g_lum_| estimated to be 2.4 × 10^−3^ and 2.3 × 10^−3^, respectively. In the same year, benzimidazole-fused [5]carbohelicene (**H4**) and protonated (**H5**) were synthesized by them [79]. Although **H4** and **H5** had the similar ϕ_F_ value about 6.0%, the fluorescence color changed from yellow (**H4**) to red (**H5**) due to the protonation process, and the |g_lum_| values were 9.45 × 10^−3^ and 5.92 × 10^−3^, respectively. This was the first observation of red-colored CPL of a helicene derivative. To further expand the chiroptical properties to the visible and near infrared region spectrum, Crassous et al. [80] reported the synthesis and chiroptical properties of π-conjugated diketopyrrolopyrrole–helicene derivative (**H6**), without using the traditional methods of metalation or functionalization by electron push–pull groups. The |g_lum_| value was 5.92 × 10^−3^, while the ϕ_F_ value had been up to 41.0%.

Another class of helicenes is the heterohelicenes, where a heteroatom (e.g., O, S, N, Si etc) inserted into the fused ring system. Heterohelicenes are generally based on five-membered rings such as thiophene, pyrrole, furan, etc., and six-membered rings mostly containing pyridine. Additionally, they can be fused and functionalized. Insertion of heteroatom in fused ring system is expected to contribute reasonably high HOMO energy level. This unique feature is highly attracted the researcher’s attention for further improvement in many fields of material sciences including OLED materials, asymmetric catalysis, chiral molecular recognition, molecular mechines, liquid crystals and so on. The structures **H7–H27** in Table 4 are heterohelicenes, respectively. Tanaka et al. [81] achieved the enantioselective synthesis of S-shaped double azahelicenes (**H7**) and (**H8**) via the Au-catalyzed sequential intramolecular hydroarylation of alkynes. Interestingly, the CPL activity of the S-shaped double azahelicenes was significantly higher than that of the azahelicenes. And the |g_lum_| values had been up to 2.8 × 10^−2^ and 1.1 × 10^−2^, respectively. Single azahelicenes, such as 3-(2-pyridyl)-4-aza[6]helicene (**H9**) [82] and 3-(2-pyridyl)-4-aza[6]-helicene (**H10**) [83], had the similar ϕ_F_ values to those of **H7** and **H8**, but had the lower |g_lum_| values than those of **H7** and **H8**. According to the results of Hiroto and Shinokubo et al. [84], the 1,4-diketones were converted to oxahelicenes (**H11**), which exhibited strong fluorescence (66.0%) both in solution and solid state as well as chiroptical properties (1.2 × 10^−3^). Tanaka et al. [85] achieved the first enantioselective synthesis of a sila[7]helicene (**H12**) through the double [2 + 2 + 2] cycloaddition of a biaryl-linked tetrayne with a silicon-linked *bis*(propargylic alcohol) as a key step. The obtained 1,1-bitriphenylene-based sila[7]helicene exhibited a high |g_lum_| value of 1.6 × 10^−2^. To examine whether and to what extent the presence of hetero-atoms in the helicene backbone could promote a CPL response, Villani et al. [34] studied a hetero [6]-helicene (**H13**) containing two sulfur atoms in the helicene backbone, and its CPL spectra were discussed on the basis of DFT calculation results. Araki et al. [86] synthesized the tetrasulfone[9]helicene (**H14**) to improve and evaluate its fluorescence and excited-state dynamics through a single-step oxidation reaction of tetrathia[9]helicene. The introduction of sulfone units onto the helicene skeleton contributed to the highly fluorescent characteristic when compared to the fluorescence of the parent thiahelicene, but the |g_lum_| value was very low, only 8.3 × 10^−4^. Vauthey et al. [87] studied the physicochemical properties of cationic dioxa (**H15**), azaoxa (**H16**), and diaza (**H17**) [6]helicenes. The fluorescence of these cationic chromophores was at the range from the orange to the near infrared regions. The |g_lum_| values were estimated to be 4.0 × 10^−4^, 2.1 × 10^−3^, and 1.1 × 10^−3^ at 595, 614, and 658 nm with the ϕ_F_ values 12.0%, 22.0% and 31.0%, respectively. Similarly, Lacour et al. [88] found the chiroptical switching properties of a water-soluble diaza [4]helicene (**H18**). This zwitterionic dye displayed pH-dependent absorption and emission properties and this enabled a reversible turn on/off of electronic CD at 300 nm and of CPL in the red region upon protonation/deprotonation. Hatakeyama et al. [89] developed a boron-fused double [5]helicenes (**H19**) possessing two boronate substructures at the ring junction synthesized from hexabromobenzene in two steps via Hart reaction and demethylative cyclization. The double helicenes showed deep blue fluorescence and CPL activity. Although its ϕ_F_ value had been up to 65.0%, its |g_lum_| value was still at the level of 10^−3^. Crassous et al. [90] synthesized the first *bis*-helicenic terpyridine ligand (**H20**) and Zn-complex (**H21**) acting as a chiroptical switch upon reversible coordination-decoordination of zinc (II). The switching process triggered conformational changes and molecular motion around the Zn center, from a trans (W-shape) conformation in the free ligand to a *cis* (U-shape) one in the Zn-complex. Compared to **H21**, **H22** increased the ϕ_F_ value to 19.0% but decreased the |g_lum_| value to 1.3 × 10^−3^. Four members of a new class of cycloborylated hexa-, octa-, and decahelicenes (**H22**–**H25**) were prepared in enantiopure form [91]. The CPL properties of these new fluorescent organic helicenes were measured and compared with the corresponding organometallic phosphorescent cycloplatinated derivatives. All the examples had the similar |g_lum_| value of 10^−3^, but mono-azabora [*n*]helicenes had the larger ϕ_F_ values (21.0% and 49.0%). Otani et al. [92] developed a facile two-step synthesis of polyazahelicenes (**H26**), which were composed of a 6-5-6-6-6-5-6 system, and showed high CPL activity under both neutral and acidic conditions (|g_lum_|: up to 9.0 × 10^−3^). Shinokubo et al. [93] synthesized a bisbutadiyne bridged azahelicene dimer with a figure-eight shape (**H27**), which exhibited a large ϕ_F_ (55.0%) and a high |g_lum_| (8.5 × 10^−3^). The enhancement was due to the rigid conformation of the dimer.

Nowadays, grafting metallic ions onto π-helical structures is not difficult and can produce novel properties. Therefore, transition metal-based helicenes have emerged as novel attractive chiral molecules. For example, organometallic helicenes with a transition metal (Pt, Ir) included in the helical π-framework as a metal center have been candidates for optoelectronic applications (OLEDs, switches, sensors, etc.). Autschbach and Crassous et al. [83] reported the first examples of rhenium-based phosphors (**H28**) and (**H29**) that exhibited CPL. By incorporating a rhenium (ReI) atom in an extended helical π-conjugated *bi*-pyridine system, the π-conjugation pathway was increased and the charge-transfer excitations within the π-helical ligand were enhanced. Compared with the neutral one **H28,** the cationic ReI complex **H29** exhibited a relatively larger ϕ_F_ (6.0%), but a little bit lower |g_lum_| (1.4 × 10^−3^). They also reported the first examples (**H30** and **H31**) of a helicene-based multiresponsive acid/base chiroptical switch based on the synthesized enantiopure rollover cycloplatinated [Pt(CH_3_)(dmso)(bipy-H)] complexes [82]. Unfortunately, both the ϕ_F_ and |g_lum_| values were smaller. Moreover, they prepared enantiopure mono-cycloplatinated-[8]helicene (**H32**) and *bis*-cycloplatinated-[6]helicene derivatives (**H33**) and (**H34**) [94]. Through the method of column chromatography and using crystallization of diastereomeric, the obtained **H33** and **H34** had the similar values of ϕ_F_ (~10.0%), but **H34** displayed a higher |g_lum_| value of the order 10^−2^. In 2017, Avarvari et al. [95] reported the first examples of chiral metal diimine dithiolene complexes. Using a platinum (II) center coordinated by 2,2’-bipyridine and helicene-dithiolene ligands, luminescent Pt(bipy) [6]helicene compound (**H35**) was synthesized. The complex was emissive in fluid solutions, and the emission band was centered at 715 nm, but the anisotropy factor was only 3 × 10^−4^. Interestingly, the Ir–NHC-helicene complexes (**H36**) and (**H37**) [96] displayed very long-lived CP blue-green phosphorescence with unusually long lifetimes and circular polarization. It depended on both the P/M stereochemistry of helical system and the D/L stereochemistry of iridium. Each pair of enantiomers displayed mirror image CP phosphorescence with the |g_lum_| of 9 × 10^−4^ for **H36** and 1.5 × 10^−3^ for **H37**, respectively.

Helicene-like refers to helical derivatives which include both aromatic or heteroaromatic and partially saturated rings in their π-conjugated scaffolds. These compounds display extremely interesting optoelectronic properties and applications in a variety of fields. Up to now, high quantum yields were only reported for helicene-like molecules in which the π-conjugation is not fully extended to the whole molecules. The structures **H38–H41** in Table 4 are helicene-like molecules, respectively. A racemic sample of 2,2′,7,7′-tetrahydroxy-1,1′-binaphthyl (**H38**) was resolved by Muller et al. [97] with (*S*)-proline and the separated enantiomers were independently converted to atropisomeric *bis*-oxazines by aromatic Mannich reaction. These chirally pure oxazines were converted to the helicene-like cyclic ethers. The CPL profile was consistent with the isolation of the targeted helical-like molecules in optically pure form, prepared from the achiral primary amines. Nozaki et al. [98] synthesized [7]helicene-like compounds with a fluorene unit (**H39**) using a platinum-catalyzed double cyclization reaction. Introduction of a fluorene substructure into a helicene framework would be the key for such a high fluorescence property and provide a promising molecular design for emissive helicenes and helicene-like compounds. Tanaka et al. [99] achieved the phenanthrenol-based [9]helicene-like molecules (**H40** and **H41**) via the rhodium-mediated intramolecular [2 + 2 + 2] cycloadditions of 3-phenanthrenol-linked triynes.

It is well known that most BODIPYs are planar and achiral, without the ability of exhibiting CPL signals, but if the BODIPY core is linked by chiral substituents or the structure of the BODIPY is modified, it can be formed into active dye exhibiting chiral properties. de la Moya’s group [100] reported a simple design, and new structures (**H42** and **H43**) were obtained by embedding dihaloBODIPY in a helically labile chiral architecture (such as flexible enantiopure diamine or diol). Knight and Hall et al. [101] reported helically chiral *N*,*N*,*O*,*O*-boron chelated dipyrromethenes (**H44**) emitting solution-phase CPL in the red region (621–663 nm). Nabeshima′s group [102] reported the synthesis of a macrocyclic *bis*BODIPY (*bis*(boron-dipyrromethene)) complex (**H45**) with a figure-of-eight helicity. The large |g_lum_| of 9 × 10^−3^ proved that **H45** was one of the most efficient red-emitting CPL emitter. They also reported a helically chiral BODIPY with a hitherto *N*,*N*,*O*,*C*-boron-chelation motif (**H46**) [103]. Synthesized by a one-pot boron metathesis, nucleophilic aromatic substitution (SNAr), Suzuki coupling, boron chelation, cascade reaction, **H46** was the first example emitting CPL from a non-C_2_-symmetric helically chiral *N*,*N*,*O*,*C*-BODIPY. The above researches provide a valuable benchmark for future developments in this compound series.

Moreover, Chen et al. [104,105] had conveniently synthesized five pairs of optically stable helical aromatic imides **H47** containing different electron donating or withdrawing groups. It was found that the enantiomers exhibited medium to high fluorescence quantum yields and full-color emissions, which represented the first examples of chiral organic molecules with full-color CPL. On the other hand, they prepared a series of enantiopure π-extended 1,16-diphenyl-3,14-diaryltetra-hydrobenzo[5]helicenediol derivatives (Ar-H[5]HOL) (**H48**) [106] by Suzuki-Miyaura cross-coupling reactions starting from 7-methoxytetralone. Also they synthesized five pairs of helical aromatic esters (**H49**) [107] with different electron-donating or electron-withdrawing groups. The emission spectra of the enantiomers not only were in the blue-color region, but also exhibited bright blue fluorescence with relatively high fluorescence quantum yields in solution and films. Moreover, the enantiomers all showed intense CPL signals with relatively high |g_lum_|.

Compared with the other chiral SOMs, more helical chiral molecules have been designed, synthesized and analyzed. They usually exhibit longer CPL wavelengths, even at 715 nm. The maximum quantum yield was obtained from **H44** (ϕ_F_ = 73.0%) and the maximum dissymmetry factor was obtained from **H7** (|g_lum_| = 2.8 × 10^−2^). Obviously, helical chiral molecules are the representatives of SOMs suitable for the visible and near infrared region CPL spectrum.

## 4. Conclusions

Recently, the design and synthesis of novel CPL materials has attracted significant attention due to the potential applications of these materials in bio-sensors, liquid crystal lasers, optical storage devices, and 3D optical displays. Organic chiral molecules are some of the most promising candidate materials for uses in advanced electronic CPL devices due to their tailored synthetic feasibility, low cost, high flexibility and processability. Therefore, it remains a relatively potential field to explore. In this review, we summarized the developments of the CPL researches on small organic molecules in the latest five years.

It can be seen that many studies have been focused on the design and synthesis of the chiral SOMs, controlling their CPL properties. For example, using designs based on chirally-perturbed simple π-extended achiral chromophores, good CPL behavior with high dissymmetry factor and fluorescence quantum yield can be obtained. However, compared with lanthanide complexes, aggregate organic molecules and macromolecules, most non-aggregate small organic molecules still have lower dissymmetry factors. Moreover, there are some difficulties in the practical applications of SOMs, especially in fields where strong CPL signs are required to emit when SOMs are in solid state solvent. Therefore, there are many aspects needing to be studied in the future:

(1) Using helicene-like chromophores, it is easy to obtain the high |g_lum_| values from small organic molecules. However, the preparation of these molecules is usually complex, requiring asymmetric catalysis and/or chiral resolution to obtain pure-enough enantiomers, which results in low overall yields. (2) Two key parameters, the quantum yield and the dissymmetry factor, have to be simultaneously optimized. One of the main difficulties in this field is that these two parameters are intrinsically related: most of the times, the optimization of one property strongly and negatively influences the other one. Modest emission quantum yields have restricted their examination in chiral optoelectronic devices or bio-imaging. (3) For every type of application CPL over the entire visible range is an important requirement. Up to now, however, their spectral responses are mainly in the blue region, and only a few examples have displayed chiroptical properties above 600 nm. It is urgent to further expand the chiroptical properties of SOMs to the near infrared region spectrum. The reason is that the red to near infrared light (650–900 nm) is relatively transparent to biological tissue and thus advantageous to imaging. (4) Most of them were studied in solution phase and were found to have low |g_lum_| values (10^−5^–10^−2^). From solution to condensed phase, the performance becomes even worse because aggregation of chiral luminophores normally populates the nonradiative pathways and thus quenches the light emission to a great extent. Fluorescence of most SOMs almost completely quenched in solid state. Development of new chiral luminescent systems with both high emission efficiency and a large dissymmetry factor in the condensed phase has been a challenging task. (5) Looking beyond the focus of this work, one can speculate to what other applications CPL may be usefully employed. For example, a new generation of chiral luminescent security tags could be created, where an appropriate polarizer could be used to identify an item by the CPL signal generated from the optical excitation of the tag. There is evidently much future promise for the field of CPL research.

## Figures and Tables

**Table 1:** CPL and relevant photophysical properties for molecules with central chirality.

No.	Structure	Solvent	λ_exc_ (nm)	λ_lum_ (nm)	ϕ_F_ (%)	|g_lum_| (10^−3^)	Ref.	Year
**C** **1**	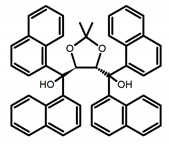	CHCl_3_ (1 × 10^−3^ M)	-	410	2.0	9.40	[44]	2014
**C** **2**	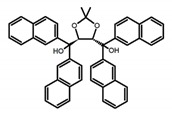	CHCl_3_ (1 × 10^−3^ M)	-	375	2.0	3.90	[44]	2014
**C3**	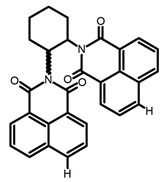	(CH_2_)_4_O (1 × 10^−5^ M)	330	450	-	14.00	[45]	2016
**C4**	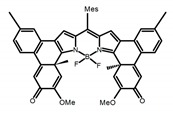	CHCl_3_ (1 × 10^−5^ M)	-	641	73.0	0.60	[46]	2016
**C5**	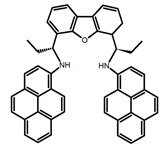	C_7_H_8_ (1 × 10^−5^ M)	-	424	60.0	0.69	[47]	2017
				500	60.0	3.90		

**Table 2:** CPL and relevant photophysical properties for molecules with axial chirality.

No.	Structure	Solvent		λ_exc_ (nm)	λ_lum_ (nm)	ϕ_F_ (%)	|g_lum_| (10^−3^)	Ref.	Year
**A1**	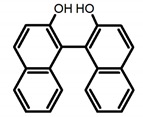	CHCl_3_ (1 × 10^−4^ M)		320	364	4.0	0.47	[48]	2015
**A2**	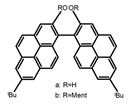	CHCl_3_ (1 × 10^−4^ M)	a	340	438	57.0	0.36	[48]	2015
			b	300	401	80.0	1.20		
**A3**	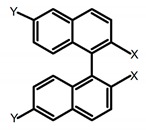 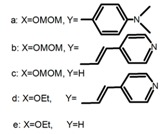	CHCl_3_ (1 × 10^−4^ M)	a	280	370	-	0.79	[49]	2018
			b	340	418	-	0.73		
			c	280	358	-	0.67		
			d	340	412	-	0.85		
			e	280	365	-	0.59		
**A4**	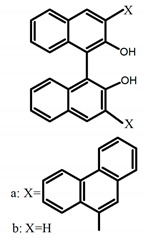	CHCl_3_ (1 × 10^−4^ M)	a	300	374	2.0	0.61	[50]	2018
			b	300	364	4.0	0.80		
		DMF (1 × 10^−4^ M)	a	320	391	38.0	0.55		
			b	320	363	6.0	0.87		
**A5**	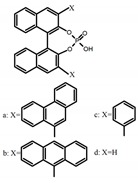	CHCl_3_ (1 × 10^−4^ M)	a	300	375	10.0	0.79	[50]	2018
			b	300	422	-	1.00		
			c	300	350	22.0	1.20		
			d	300	350	29.0	1.50		
		DMF (1 × 10^−4^ M)	a	300	373	21.0	0.32		
			b	300	415	-	0.43		
			c	300	370	22.0	1.90		
			d	300	361	28.0	1.90		
**A6**	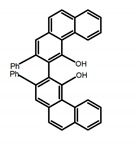	CHCl_3_ (1 × 10^−^^5^ M)		282	376	20.0	1.30	[51]	2014
		CHCl_3_ (1 × 10^−^^4^ M)		289	378	16.0	-		
		CHCl_3_ (1 × 10^−^^3^ M)		320	391	13.0	1.10		
**A7**	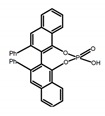	CHCl_3_ (1 × 10^−^^4^ M)		307	372	12.0	3.50	[52]	2015
		PMMA		320	372	16.0	2.00		
**A8**	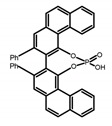	CHCl_3_ (1 × 10^−^^4^ M)		335	373	9.0	1.30	[52]	2015
		PMMA		330	376	19.0	0.79		
**A9**	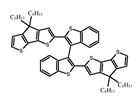	CHCl_3_ (2 × 10^−^^4^ M)		-	~520	21.0	9.30	[53]	2018
**A10**	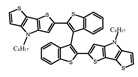	CHCl_3_ (2 × 10^−^^4^ M)		-	~500	12.0	8.00	[53]	2018
**A11**	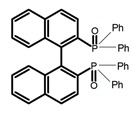	CHCl_3_ (1 × 10^−^^4^ M)		300	~355	2.0	1.20	[54]	2015
		PMMA		300	354	7.0	1.00		
		KBr pellet		330	372	7.0	0.67		
**A12**	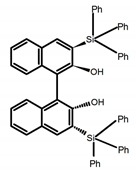	CHCl_3_ (1 × 10^−^^4^ M)		280	375	18.0	1.00	[55]	2016
		PMMA		-	368	5.0	0.61		
		KBr pellet		-	372	8.0	0.25		
**A13**	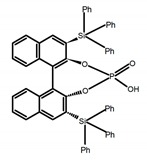	CHCl_3_ (1 × 10^−4^M)		280	363	35.0	1.30	[55]	2016
		PMMA		-	362	15.0	1.30		
		KBr pellet		-	367	29.0	0.36		
**A14**	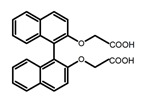	CHCl_3_ (1 × 10^−4^ M)		300	~360	25.0	0.18	[56]	2016
		DMF (1 × 10^−4^ M)		300	~375	39.0	0.19		
		CH_3_CN (1 × 10^−^^4^ M)		290	~370	29.0	0.03		
		MeOH (1 × 10^−4^ M)		290	~360	32.0	0.14		
		PMMA		300	~360	11.0	0.42		
**A15**	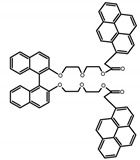	CHCl_3_ (1 × 10^−^^3^ M)		340	480	20.0	0.78	[57]	2014
		PMMA		340	~400	46.0	0.36		
**A16**	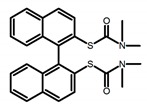	CHCl_3_ (1 × 10^−4^ M)		300	400	2.0	1.20	[58]	2018
		PMMA		300	no	4.0	no		
		ARTON		300	no	2.0	no		
**A17**	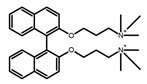	Water (1 × 10^−4^ M)		300	374	18.0	0.30	[59]	2017
**A18**	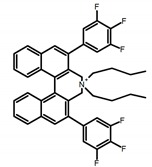	Water (1 × 10^−4^ M)		310	371	16.0	0.20	[59]	2017
**A19**	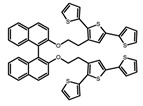	KBr pellet		297	~410	3.0	0.50	[60]	2015
**A20**	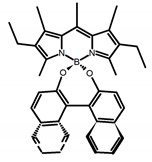	CHCl_3_ (1 × 10^−3^ M)		529	570	45.0	0.78	[61]	2014
**A21**	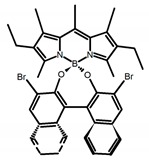	CHCl_3_ (1 × 10^−3^ M)		445	575	69.0	0.70	[62]	2017
**A22**	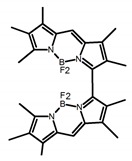	CH_2_Cl_2_ (5 × 10^−5^ M)		-	655	-	3.80	[35]	2016
**A23**	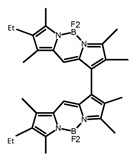	CH_2_Cl_2_ (5 × 10^−5^ M)		-	603	-	0.40	[35]	2016
**A24**	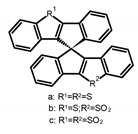	CH_2_Cl_2_ (1 × 10^−^^5^ M)	a	300	368	6.0	1.50	[63]	2017
		CH_2_Cl_2_ (6 × 10^−^^6^ M)	b	330	459	1.0	3.00		
		CH_2_Cl_2_ (1 × 10^−^^5^ M)	c	350	444	76.0	1.50		
**A25**	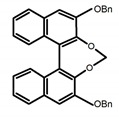	C_4_H_8_O_2_ (1 × 10^−5^ M)		310	~360	44.0	0.68	[64]	2017
		Solid state		310	~360	13.0	1.10		
**A26**	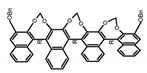	C_4_H_8_O_2_ (5 × 10^−6^ M)		330	~400	78.0	1.60	[64]	2017
		Solid state		330	~400	29.0	1.40		
**A27**	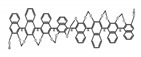	C_4_H_8_O_2_(2.5 × 10^−^^6^ M)		330	~410	90.0	2.20	[64]	2017
		Solid state		330	~410	22.0	7.00		
**A28**	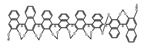	C_4_H_8_O_2_ (2.5 × 10^−^^6^ M)		330	~410	79.0	0.57	[64]	2017
		Solid state		330	~410	24.0	-		
**A29**	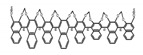	C_4_H_8_O_2_ (2.5 × 10^−^^6^ M)		330	~410	84.0	0.38	[64]	2017
		Solid state		330	~410	17.0	-		
**A30**	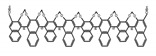	C_4_H_8_O_2_ (2.5 × 10^−^^6^ M)		330	~410	64.0	0.31	[64]	2017
		Solid state		330	~410	13.0	-		
**A31**	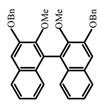	CHCl_3_ (1 × 10^−4^ M)		290	343	7.0	0.58	[65]	2018
		PMMA		290	348	5.0	0.65		
**A32**	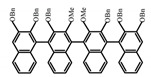	CHCl_3_ (1 × 10^−4^ M)		300	354	40.0	0.61	[65]	2018
		PMMA		300	354	16.0	0.51		
**A33**	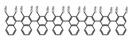	CHCl_3_ (1 × 10^−4^ M)		290	363	52.0	0.33	[65]	2018
		PMMA		305	358	29.0	0.49		

**Table 3:** CPL and relevant photophysical properties for molecules with planar chirality.

No.	Structure	Solvent	λ_exc_ (nm)	λ_lum_ (nm)	ϕ_F_ (%)	|g_lum_| (10^−3^)	Ref.	Year
**P** **1**	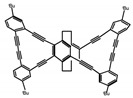	CHCl_3_ (1 × 10^−5^ M)	314	460	45.0	11.00	[66]	2014
**P** **2**	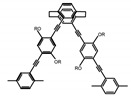	CHCl_3_ (1 × 10^−5^ M)	320	407	50.0	1.80	[67]	2014
**P3**	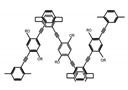	CHCl_3_ (1 × 10^−5^ M)	320	414	47.0	2.20	[67]	2014
**P4**	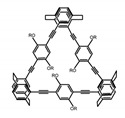	CHCl_3_ (1 × 10^−5^ M)	320	413	64.0	2.20	[67]	2014
**P5**	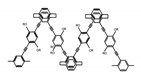	CHCl_3_ (1 × 10^−5^ M)	320	415	60.0	2.60	[67]	2014
**P6**	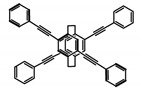	CHCl_3_ (1 × 10^−5^ M)	300	412	60.0	1.20	[68]	2015
**P7**	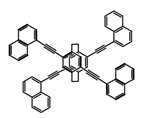	CHCl_3_ (1 × 10^−5^ M)	300	421	78.0	1.70	[68]	2015
**P8**	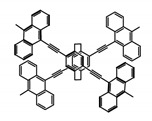	CHCl_3_ (1 × 10^−5^ M)	350	503	42.0	0.50	[68]	2015
**P9**	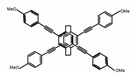	CHCl_3_ (1 × 10^−5^ M)	-	416	66.0	1.50	[69]	2016
**P10**	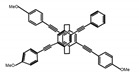	CHCl_3_ (1 × 10^−5^ M)	280	425	>70.0	1.70	[70]	2018
**P11**	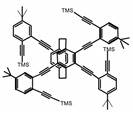	CHCl_3_ (1 × 10^−5^ M)	300	418	46.0	1.40	[71]	2015
**P12**	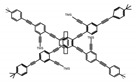	CHCl_3_ (1 × 10^−5^ M)	350	438	80.0	1.20	[71]	2015
**P13**	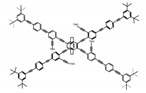	CHCl_3_ (1 × 10^−5^ M)	350	443	88.0	1.00	[71]	2015
**P14**	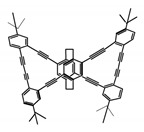	CHCl_3_ (1 × 10^−5^ M)	314	453	41.0	13.00	[71]	2015
**P15**	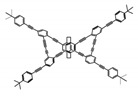	CHCl_3_ (1 × 10^−5^ M)	350	471	60.0	10.00	[71]	2015
**P16**	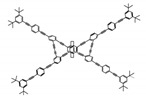	CHCl_3_ (1 × 10^−5^ M)	355	474	70.0	7.50	[71]	2015
**P17**	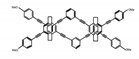	CHCl_3_ (1 × 10^−5^ M)	379	419	62.0	1.60	[72]	2016
**P18**	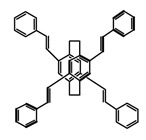	C_4_H_8_O_2_ (1 × 10^−5^ M)	350	455	78.0	3.70	[73]	2017
**P19**	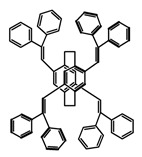	C_4_H_8_O_2_ (1 × 10^−5^ M)	350	494	58.0	0.73	[73]	2017
**P20**	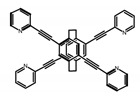	CH_2_Cl_2_ (1 × 10^−5^ M)	300	421	59.0	2.80	[74]	2017
**P21**	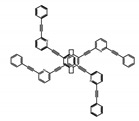	CH_2_Cl_2_ (1 × 10^−5^ M)	300	417	56.0	1.20	[74]	2017
**P22**	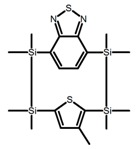	Cyclohexane (1.5 × 10^−5^ M)	370–380	500	5.0	1.70	[75]	2017
**P23**	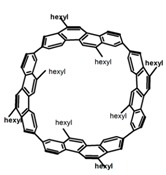	C_7_H_8_ (4.90–6.77 × 10^−6^ M)	-	443	80.0	15.00	[76]	2017
**P24**	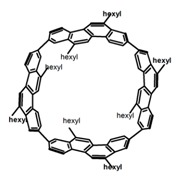	C_7_H_8_ (4.90–6.77 × 10^−6^ M)	-	~450	74.0	10.00	[76]	2017

**Table 4:** CPL and relevant photophysical properties for molecules with helical chirality.

No.	Structure	Solvent		λ_exc_ (nm)	λ_lum_ (nm)	ϕ_F_ (%)	|g_lum_| (10^−3^)	Ref.	Year
**H** **1**	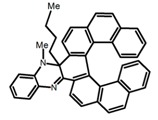	(CH_2_)_4_O		330	~550	25.0	4.00	[77]	2015
**H** **2**	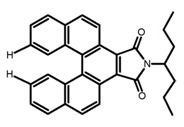	(CH_2_)_4_O		-	~480	37.0	2.40	[78]	2016
**H3**	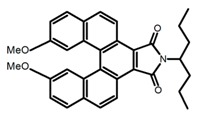	(CH_2_)_4_O		-	~480	22.0	2.30	[78]	2016
**H4**	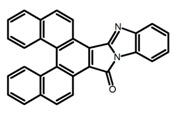	CH_2_Cl_2_		-	550	~6.0	9.45	[79]	2016
**H5**	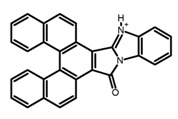	CH_2_Cl_2_		-	650	~6.0	5.92	[79]	2016
**H6**	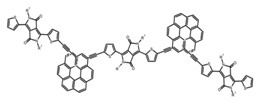	CH_2_Cl_2_		-	610	41.0	0.30	[80]	2018
**H7**	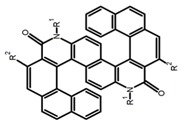	CHCl_3_ (1 × 10^−6^ M)		375	492	19.0	28.00	[81]	2014
**H8**	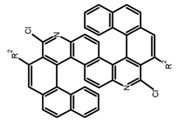	CHCl_3_ (1 × 10^−6^ M)		375	454	9.0	11.00	[81]	2014
**H9**	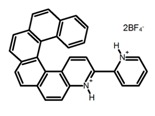	CH_2_Cl_2_		-	590	8.0	2.90	[82]	2015
**H10**	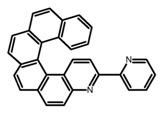	CH_2_Cl_2_		-	421	8.0	3.20	[83]	2015
**H11**	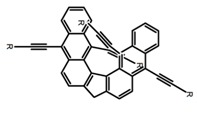	CH_2_Cl_2_ (1 × 10^−5^ M)		-	~550	66.0	1.20	[84]	2015
**H12**	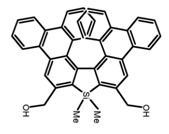	CHCl_3_		-	482	15.0	16.00	[85]	2015
**H13**	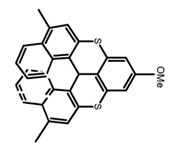	CHCl_3_ (2 × 10^−4^ M)		390	~500	1.6	9.00	[34]	2016
**H14**	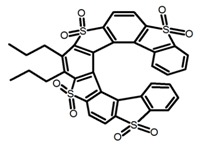	C_7_H_8_		-	~430	27.0	0.83	[86]	2016
**H15**	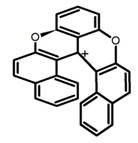	CH_2_Cl_2_ (2 × 10^−3^ M)		435	595	12.0	0.40	[87]	2016
**H16**	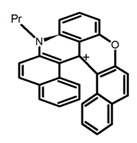	CH_2_Cl_2_ (2 × 10^−3^ M)		442	614	22.0	2.10	[87]	2016
**H17**	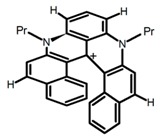	CH_2_Cl_2_ (2 × 10^−3^ M)		473	658	31.0	1.10	[87]	2016
**H18**	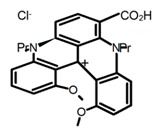	CH_3_CN (1 × 10^−5^ M)		-	654	29.0	0.50	[88]	2016
**H19**	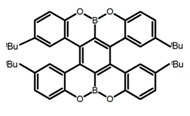	CH_2_Cl_2_ (2 × 10^-3^ M)		-	436	65.0	1.70	[89]	2016
**H20**	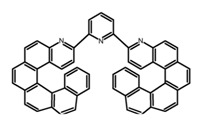	CH_2_Cl_2_ (2 × 10^−3^ M)		-	421	8.4	8.50	[90]	2016
**H21**	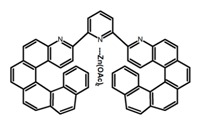	CH_2_Cl_2_ (2 × 10^−3^ M)		-	480	19.0	1.30	[90]	2016
**H22**	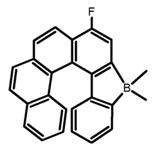	CH_2_Cl_2_		395	430	21.0	0.90	[91]	2017
**H23**	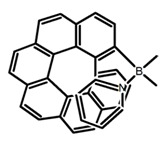	CH_2_Cl_2_		405	430	49.0	2.30	[91]	2017
**H24**	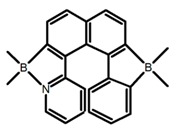	CH_2_Cl_2_		405	442	7.0	0.70	[91]	2017
**H25**	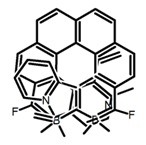	CH_2_Cl_2_		420	473	7.0	1.00	[91]	2017
**H26**	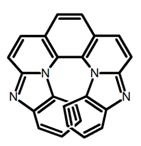	CH_2_Cl_2_ (1 × 10^−5^ M)		380	473	39.0	9.00	[92]	2017
**H27**	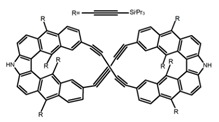	CH_2_Cl_2_		-	588	55.0	8.50	[93]	2017
**H28**	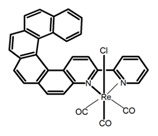	CH_2_Cl_2_ (1 × 10^−3^ M)		-	673	0.2	3.00	[83]	2015
**H29**	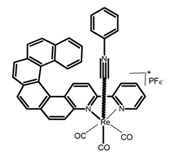	CH_2_Cl_2_ (1 × 10^−3^ M)		-	598	6.0	1.40	[83]	2015
**H30**	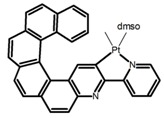	CH_2_Cl_2_		-	547	0.4	1.10	[82]	2015
**H31**	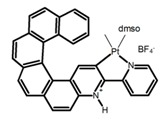	CH_2_Cl_2_		-	555	3.0	2.00	[82]	2015
**H32**	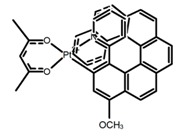	CH_2_Cl_2_ (1 × 10^−3^ M)		452	648	5.6	4.50	[94]	2014
**H33**	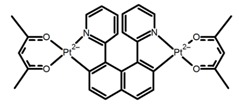	CH_2_Cl_2_(1 × 10^−3^ M)		459–469	633	13.0	0.50	[94]	2014
**H34**	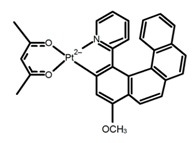	CH_2_Cl_2_ (1 × 10^−3^ M)		452	644	10.0	12.00	[94]	2014
**H35**	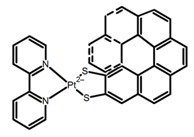	CH_3_CN (3 × 10^−4^ M)		-	715	15.0	0.30	[95]	2017
**H36**	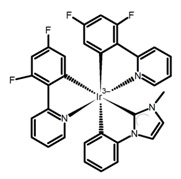	CH_2_Cl_2_ (5 × 10^−5^ M)		-	493	-	0.90	[96]	2017
**H37**	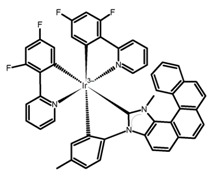	CH_2_Cl_2_ (5 × 10^−5^ M)		-	530	9.0~ 13.0	1.50	[96]	2017
**H38**	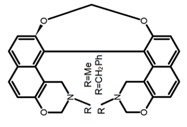	CH_3_CN (1 × 10^−3^ M)		357	-	-	~1.30	[97]	2014
**H39**	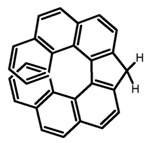	CH_2_Cl_2_ (1 × 10^−5^ M)		-	417	39.0	3.00	[98]	2016
**H40**	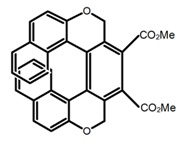	CHCl_3_ (2 × 10_−5_ M)		350	473	23.0	0.95	[99]	2017
**H41**	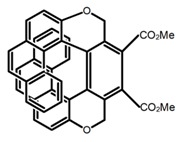	CHCl_3_ (2 × 10^−5^ M)		350	547	18.0	1.10	[99]	2017
**H42**	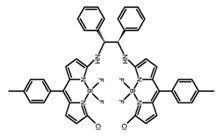	CHCl_3_ (1 × 10^−3^ M)		-	~570	14.0	1.00	[100]	2016
**H43**	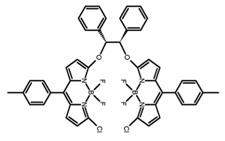	CHCl_3_ (1 × 10^−3^ M)		-	~550	16.0	1.00	[100]	2016
**H44**	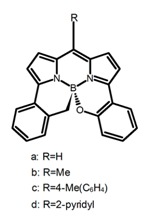	MeCN	a	540	623	65.0	4.70	[101]	2016
			b		635	73.0	3.30		
			c		637	52.0	4.30		
			d		675	28.0	4.20		
**H45**	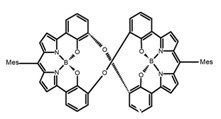	CHCl_3_ (1 × 10^−5^ M)		400	663	58.0	9.00	[102]	2016
**H46**	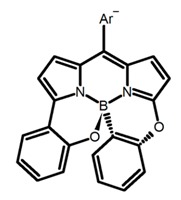	C_6_H_14_		-	622	49.0	3.70	[103]	2017
**H47**	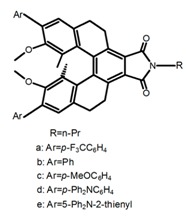	(CH_2_)_4_O (1 × 10^−5^ M)	a	366	445	13.0	~1.00	[104]	2016
			b	366	457	19.0	1.45		
			c	371	482	65.0	~1.25		
			d	387	556	40.0	~0.80		
			e	415	617	7.0	0.30		
		Water (2.5 × 10^−5^ M)	a	-	463	12.0	1.70	[105]	2018
			b	-	478	13.0	1.10		
			c	-	492	21.0	1.30		
			d	-	545	16.0	0.70		
			e	-	603	3.0	0.10		
**H48**	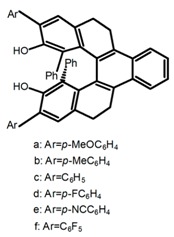	CH_2_Cl_2_ (1 × 10^−5^ M)	a	-	408	27.0	0.89	[106]	2017
			b	-	407	33.0	0.90		
			c	-	409	23.0	1.00		
			d	-	406	35.0	0.61		
			e	-	416	22.0	0.77		
			f	-	408	27.0	0.76		
**H49**	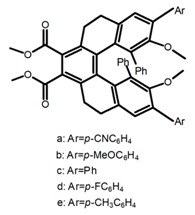	(CH_2_)_4_O (5 × 10^−5^ M)	a	-	459	48.0	5.33	[107]	2017
			b	-	453	59.0	3.53		
			c	-	453	40.0	6.08		
			d	-	459	41.0	6.50		
			e	-	453	42.0	4.63		
